# Enhancing the biotransformation of progesterone to the anticancer compound testololactone by *Penicillium chrysogenum* Ras3009: kinetic modelling and efficiency maximization

**DOI:** 10.1186/s12896-024-00896-9

**Published:** 2024-10-04

**Authors:** Marwa M. Abdel-Kareem, Abdel-Nasser A. Zohri, Abdel-Hamied M. Rasmey, Heba Hawary

**Affiliations:** 1 Department of Botany and Microbiology, Faculty of Science, Sohag University, Sohag, EG-82524 Egypt; 2 Department of Botany and Microbiology, Faculty of Science, Assiut University, Assiut, EG-71516 Egypt; 3 Department of Botany and Microbiology, Faculty of Science, Suez University, P.O.Box: 43221, Suez, Egypt

**Keywords:** Pharmaceutical compounds, Steroids biotransformation, Testololactone, Progesterone, Kinetic modelling, GC-MS analysis

## Abstract

**Background:**

Biotransformation of steroid compounds into therapeutic products using microorganisms offers an eco-friendly and economically sustainable approach to the pharmaceutical industry rather than a chemical synthesis way. The biotransformation efficiency of progesterone into the anticancer compound testololactone using *Penicillium chrysogenum* Ras3009 has been investigated. Besides, maximization of testololactone formation was achieved by studying the kinetic modelling and impact of some fermentation conditions on the biotransformation process.

**Results:**

The fungal strain Ras3009 was selected among twelve fungal strains as the most runner for the transformation of 81.18% of progesterone into testololactone. Ras3009 was identified phenotypically and genotypically as *Penicillium chrysogenum*, its 18 S rRNA nucleotide sequence was deposited in the GenBank database by the accession number *OR480104.* Studying the impact of fermentation conditions on biotransformation efficiency indicated a positive correlation between substrate concentration and testololactone formation until reaching the maximum velocity *v*_*max*_. Kinetic studies revealed that *v*_*max*_ was $\:0.0482$ gL^− 1^hr^− 1^ with high accuracy, giving *R*^2^ of 0.977. The progesterone transformation efficiency generally increased with time, reaching a maximum of 100% at 42 h with testololactone yield (*Y*_*pt/s*_) 0.8700 mg/mg. Moreover, the study indicated that the enzymatic conversion by *P. chrysogenum* Ras3009 showed high affinity to the substrate, intracellularly expressed, and released during cell disruption, leading to higher efficiency when using whole microbial cell extract.

**Conclusions:**

Fungi can be promising biocatalysts for steroid transformation into valuable chemicals and pharmaceutical compounds. The study revealed that the new fungal isolate *P. chrysogenum* Ras3009 possesses a great catalytic ability to convert progesterone into testololactone. Kinetic modelling analysis and optimization of the fermentation conditions lead to higher transformation efficiency and provide a better understanding of the transformation processes.

**Graphical abstract:**

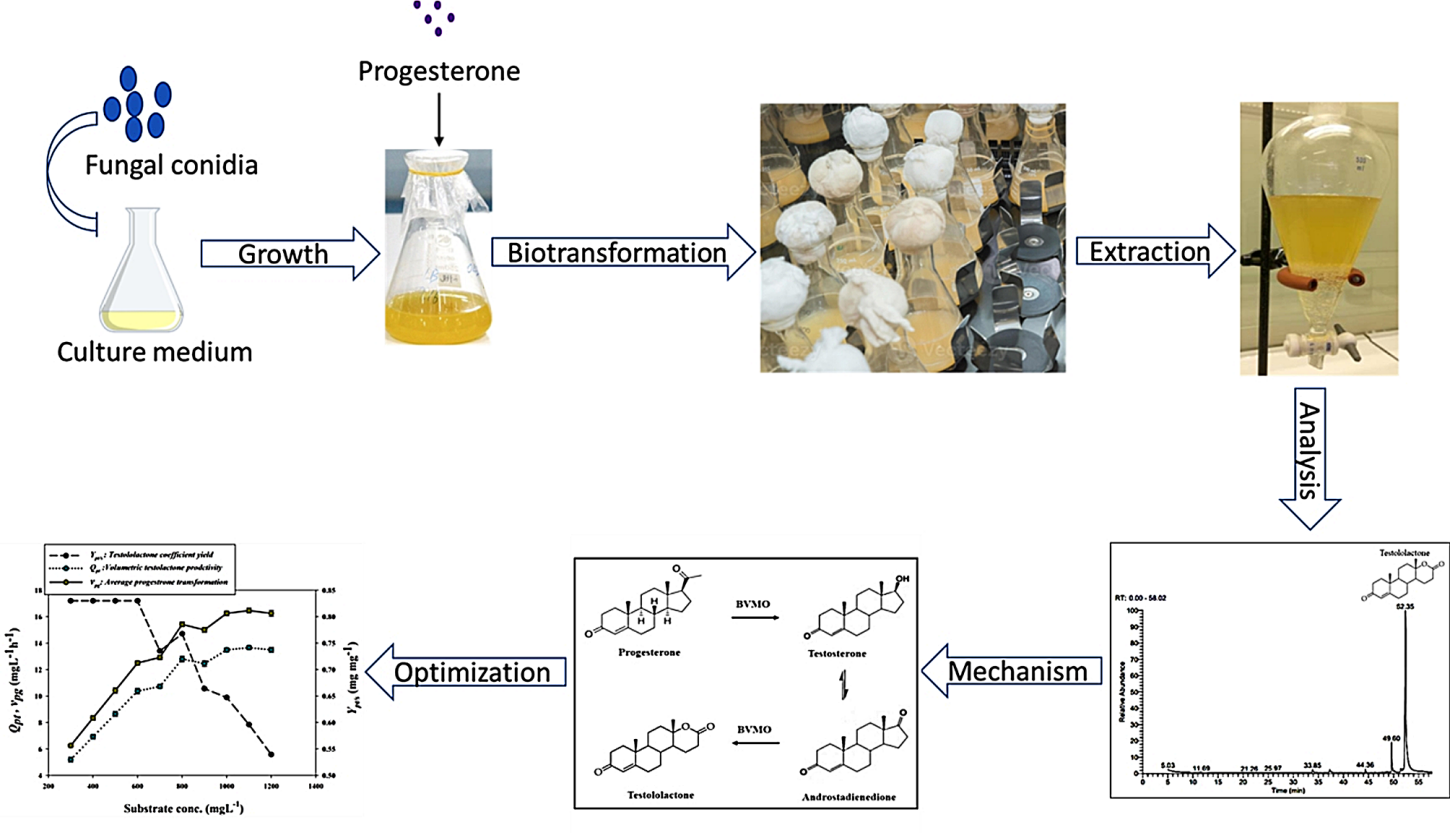

## Background

The term biotransformation refers to the chemical modification of compounds by living organisms, it refers to any enzyme-catalyzed reaction, including reactions using cell extracts or isolated enzymes [1, 2]. Biotransformation by different isolated microorganisms or enzymes has been widely used for the biotransformation of steroid molecules, thus becoming one of the best examples of a sustainable approach to applying this strategy [3, 4]. Steroid compounds are arguably one of the most widely marketed products in the pharmaceutical industry and the second largest group of medicines used to treat many diseases [5]. The biological activity of these compounds depends mainly on their chemical structure and the position of the functional groups [6]. The manufactured steroidal derivatives are widely used as anti-inflammatory, immunosuppressive, diuretic, anabolic, anti-androgenic, progestogenic, and contraceptive agents [7–9]. They are also used successfully to treat some forms of cancer, like breast cancer and prostate cancer, to prevent coronary heart disease, and as antifungal agents, as well as prevent and treat HIV infection and declared AIDS [10–12].

Due to the complexity of the steroid molecule, highly specific reactions are required to produce functionalized compounds with therapeutic use and commercial value [13, 14]. Steroid drugs are synthesized via chemical or microbial pathways, both of which involve the conversion of steroid precursors into drug intermediates and the final conversion of intermediates into active drugs [15–17]. Compared with chemical synthesis methods, high-yield biological production methods are more eco-friendly and have a greater economic impact on the production process, this feature is currently a major concern for industrialists. The biotransformation process is one of the most common biological methods for producing steroid derivatives [14].

 [14].

Nowadays, steroid drug production by microorganisms is one of the most successful applications of microbial technology in the industry sector in large-scale processes [18]. A wide range of bacterial and fungal species can transform several steroid compounds into commercial therapeutic products. Fungi are preferred among other microorganisms for the biotransformation of a wide range of steroids due to their ability to secrete numerous multipurpose enzymes [14]. The first realization of the importance of microbial biotechnology in the production of steroid drugs was in 1952 by Murray and Peterson, where progesterone was transformed into 11α-hydroxyprogesterone by *Rhizopus* species as the first recorded microbial transformation process of steroids [19]. Subsequently, some fungi have been used as microbial biocatalysts for many biotransformation reactions of steroids, especially hydroxylation reactions, and side-chain degradation reactions of progesterone [18]. Microbial cleavage of steroid side chains has been the subject of extensive research due to the importance of this reaction for the production of sex hormones and anticancer compounds from cheap and available natural steroid substrates [10, 20].

A variety of fungal species, especially the species belonging to the two genera *Aspergillus* and *Penicillium* can degrade and cleave the side chain of progesterone ending with several medicinal products [21, 22]. During side chain degradation of progesterone, oxygen is typically introduced between C-17 and C-20 to form 17-acetate, which is then cleaved by esterases producing testosterone (17-hydroxysteroid). The 17-hydroxyl is then oxidized to 17-keto which is subjected to a 1-dehydrogenation reaction ending in Androst-4-ene-3,17-dione [AD] and Androst-1,4-diene-3,17-dione [ADD]. Through further chemical modification, it can be used to produce androgens, estrogens, and other compounds that are approved for the treatment of breast cancer [23].

Testololactone is an antineoplastic drug used to treat advanced breast cancer as a steroidal aromatase inhibitor. It is generally used to treat various breast cancers in menopausal women or those who have lost ovarian function. It works by blocking the production of estrogen, thereby helping to prevent the growth of estrogen-stimulated breast cancer. It also may prevent tumor cells from being activated by other hormones [24].

Several previous studies stated that the biotransformation process refers only to reactions inside the microbial cells. However, in unicellular and filamentous fungi, it is often difficult to distinguish whether a reaction occurs intracellularly or is catalyzed by secreted extracellular enzymes or by enzymes secreted both intra- and extracellular. Recently, various metabolic pathways of steroid transformation by microorganisms have been studied to declare the mechanisms of transformation and to enhance productivity [9]. So, our interest lies in preparing new steroids that are chemically difficult to synthesize, maximizing the biotransformation efficiency, and increasing the yield of the formed product. Consequently, this study aimed to biotransform progesterone into testololactone as an anticancer compound using the fungal strain *Penicillium chrysogenum* Ras3009. Subsequently, the bioconversion efficiency was maximized by studying the effects of some fermentation conditions and kinetic modelling analysis.

## Materials and methods

### Source of microorganisms

Twelve tested fungal isolates in this study were isolated by the pour plate method from cultivated soil samples collected from Assiut governorate, Egypt. Potato dextrose agar (PDA) was used as isolation media consisting of (g/L): potato extract 4 (from 200 g infused potato), dextrose 20, and agar 20 having pH 5.6. The picked-up pure colonies were purified on PDA plates and then preserved at 4 °C. The isolated fungal isolates were used in the progress experiments for progesterone biotransformation.

### Fungal inocula preparation

The isolated fungal inoculum was prepared by inoculating 50 ml of sterile PD broth medium at pH 5.5 with one ml spore suspension from a five-day-old culture. The inoculated broth medium was incubated at 28 °C and 150 rpm for 5 days. All inocula used for biotransformation experiments were adjusted to 1 × 10^6^ spores/ml.

### Biotransformation process

The biotransformation medium used was Czapek’s broth consisting of (g/L): glucose 10.0, NaNO_3_ 3.0, KH_2_PO_4_ 1.0, MgSO_4_·7H_2_O 0.5, KCl 0.5, and FeSO_4_·7H_2_O 0.01 with pH 5.5. Each 50 ml sterilized medium in an Erlenmeyer flask (250 ml) was inoculated with 2.5 mL (5%) spore suspension (1 × 10^6^ spores/ml) and was incubated in a shaking incubator at 150 rpm, 28 ± 2 °C for 48 h until fungal pellets formed. Then, 25 mg of progesterone dissolved in 1 ml of 96% ethanol was added to the fermentation medium under aseptic conditions and continued incubation for another 48 h for the transformation process. Parallel controls without progesterone or a microorganism were also considered.

### Extraction of transformed products

After fermentation, the transformed products and residual progesterone were extracted by mixing the contents of each culture with double its volume of chloroform (100 ml) and homogenized using an ultrasonic sonicator to disturb the cell walls. The homogenized chloroform extracts were filtered, and chloroform layer containing the transformed products was separated by a separation funnel. The separated chloroform layer was filtered on anhydrous sodium bicarbonate and then dried in a rotary evaporator at 45 °C.

### GC-MS analysis of transformed products

The residual crude extract was dissolved in 1 mL chloroform, filtered through a 0.2 μm syringe filter, and used for gas chromatography-mass spectrometry analysis with the following specifications. Instrument: TRACE GC Ultra gas chromatograph (THERMO Scientific, USA) equipped with a THERMO mass spectrometer detector (ISQ single quadrupole mass spectrometer). The GC-MS system was equipped with a TG-5MS column (30 m x 0.32 mm i.d., 0.25 μm film thickness). The analysis was performed with helium as the carrier gas at a flow rate of 1.0 ml/min. The temperature program was as follows: 50 °C for 1 min; ramp to 280 °C at 5 C/min and hold for 5 min. The temperatures of the injector and detector are 220 °C and 200 °C respectively. One µL of diluted sample (1:10 diethyl ether, v/v) was injected. Mass spectra were obtained by 70 eV electron ionization (EI) with a spectral range of m/z 50–500. Chemical identification of compounds was based on a comparison of their mass spectra with those in the NIST mass spectral library and a comparison of their retention indices with standard compounds.

### Morphological characterization and genotypic identification of the fungal isolate Ras3009

#### Morphological characterization

The macroscopic and microscopic morphological growth characteristics of the selected fungal isolate Ras3009 were carried out on PDA medium according to Visagie et al. [25].

#### Genotypic identification

##### DNA extraction and PCR amplification

Mycelial pellets of the fungal isolate Ras3009 were obtained by inoculating potato dextrose broth medium with a spore suspension of the fungus and incubated at 28 °C for 5 days. DNA was extracted from the fungus pellets using extraction kits according to the method of Rasmey et al. [26]. Polymerase chain reaction (PCR) amplification of 18 S rRNA genes was performed on the extracted fungal DNA using the Qiagen Proof-Start Tag Polymerase Kit (Qiagen, Hilden, Germany). The used two primers were: 18SF: 5/-TTAAGCCATGCATGTCTAAG-3/ (forward) and 18SR: 5/-GACTACGACGGTATCTAATC-3/ (reverse). A total reaction volume of 25 µl was used, including 50 ng template DNA, 12.5 µl PCR Master Mix, 5 pmol (0.5 µl) of each primer, and completed with 11.5 µl DNase-free water. The reaction mixture was incubated in an automated thermal cycler (Master cycler, Eppendorf, Germany) under the following conditions: 35 cycles of DNA denaturation at 94 °C for 30 s, followed by annealing at 52 °C for 30 s, and extension at 72 °C for 3 min. The reaction was stopped by freezing at 4 °C. PCR products were analyzed by electrophoresis on a 1% (v/v) agarose TBE gel (tribasic boron ethylenediaminetetraacetic acid gel) and were visualized under UV light. PCR products were purified from the gel using the QIA Rapid Gel Extraction Kit (Qiagen, Hilden, Germany).

### DNA sequencing

The amplified PCR bands were sequenced bidirectionally using an automated DNA sequencer (3500 Genetic Analyzer, Applied Biosystems). Using Blast search to determine the similarity degree, the nucleotide sequence obtained was consistent with that previously published on the NCBI website (http://www.ncbi.nlm.nih.gov/BLAST/). Multiple sequence analysis and MP (maximum resolution) plots were performed using CLUSTALX (http://clustalw.ddbj.nig.ac.jp/top-ehtml) and the software MEGA 7.2.2. Also, phylogenetic tree construction of the 18 S rRNA gene sequence of isolate Ras2003 with other related sequences in the GenBank database was performed using MEGA 7 [27]. The evolutionary history was analyzed using the Neighbor-Joining method [28]. Evolutionary distances were calculated using the maximum composite likelihood method [29].

### Optimization of the biotransformation process

To maximize the biotransformation efficiency into testololactone, the effects of some factors were studied, like substrate concentration, incubation period, and cell partition extract. The effect of substrate (progesterone) concentration was detected by adding different concentrations of progesterone (300–1200 mg/L) individually. Also, the effect of the incubation period was studied by incubating the culture under the biotransformation process after the addition of progesterone at different incubation times (0–55 h). In addition, the cell partition extract effect was tested by extraction of intracellular, extracellular, and whole-cell (intracellular and extracellular) transformed testololactone individually, and the products were qualitative and quantitatively determined. For each experiment, the transformation efficiency (*R*_*E*_), testololactone concentrations (*P*_*t*_), testololactone coefficient yield (*Y*_*pt/s*_), volumetric testololactone production rate (*Q*_*pt*_), and average progesterone transformation rate (*v*_*pg*_) were calculated based on the following equations: the transformation efficiency was calculated based on the percent of the spent nontransformed progesterone (*S*_*nt*_) in respect to the initial progesterone concentration (*S*_*go*_) [Eq. 1], testololactone coefficient yield was determined based on the initial progesterone concentration (*S*_*go*_) and expressed in mg/mg [Eq. 2], volumetric testololactone productivity was calculated relying on the testolactone concentration (*P*_*t*_*)* (mg L^− 1^) during the incubation time t (h) giving the highest testololactone concentration [Eq. 3] while the average progesterone transformation rate (*v*_*pg*_) was measured as milligrams of progesterone transformed (*S*_*t*_) per liter per hour (mgL^− 1^h^− 1^) [Eq. 4].

1 $${R_E}\left( \% \right) = 100\left( {{S_{nt}}/{S_{g0}}} \right)$$

2 $${Y_{pt/s}}\left( {mg\,m{g^{ - 1}}} \right) = {P_t}/{S_{g0}}$$

3 $${Q_{pt}}\left( {mg{L^{ - 1}}{h^{ - 1}}} \right) = d{P_t}/dt$$

4 $${v_{pg}}\left( {mg{L^{ - 1}}h{r^{ - 1}}} \right) = - d{S_g}/dt$$

### Statistical analysis

Progesterone transformation parameters mean values were compared at a 5% significance level using Tukey’s test. Non-linear regression analysis was performed using the SPSS 10 statistical program.

## Results

**Fig.1:**
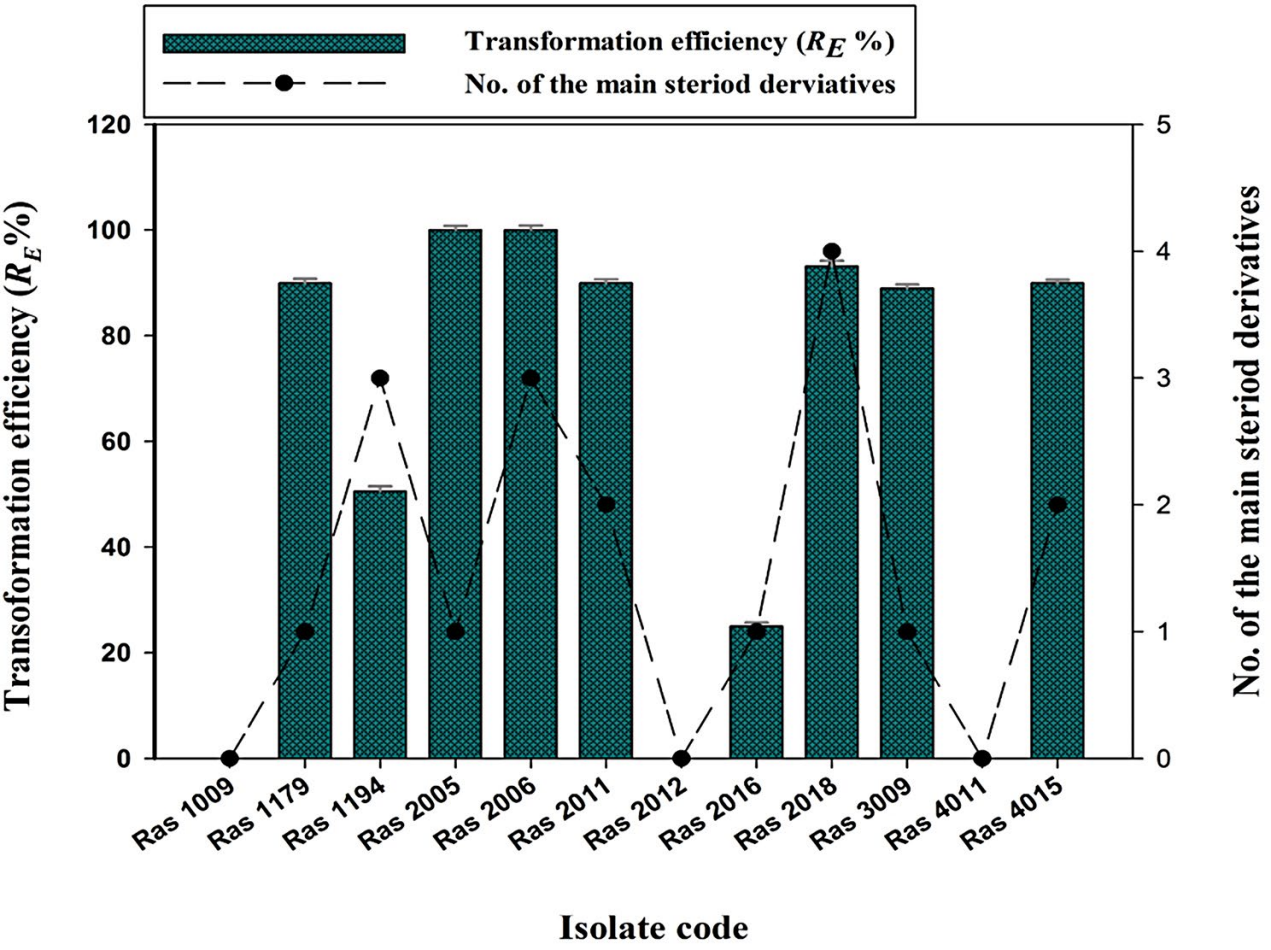
The progesterone transformation efficiency (%) of the tested fungal isolates

Twelve fungal isolates recovered from cultivated soil can convert progesterone into other derivatives, especially testololactone with high yields. The results in Fig. 1 indicated that among twelve tested fungal isolates, two isolates, Ras2006 and Ras2005, exhibited 100% transformation efficiency, which means that they fully converted progesterone into other derivatives. Another five isolates (Ras1179, Ras2011, Ras2018, Ras3009, and Ras4015) were able to transform about 89 to 93% progesterone into other derivatives. However, the remaining five isolates demonstrated lower transformation efficiencies, ranging from 0 to 50.57%, indicating that they were less effective in converting progesterone.

**Fig.2:**
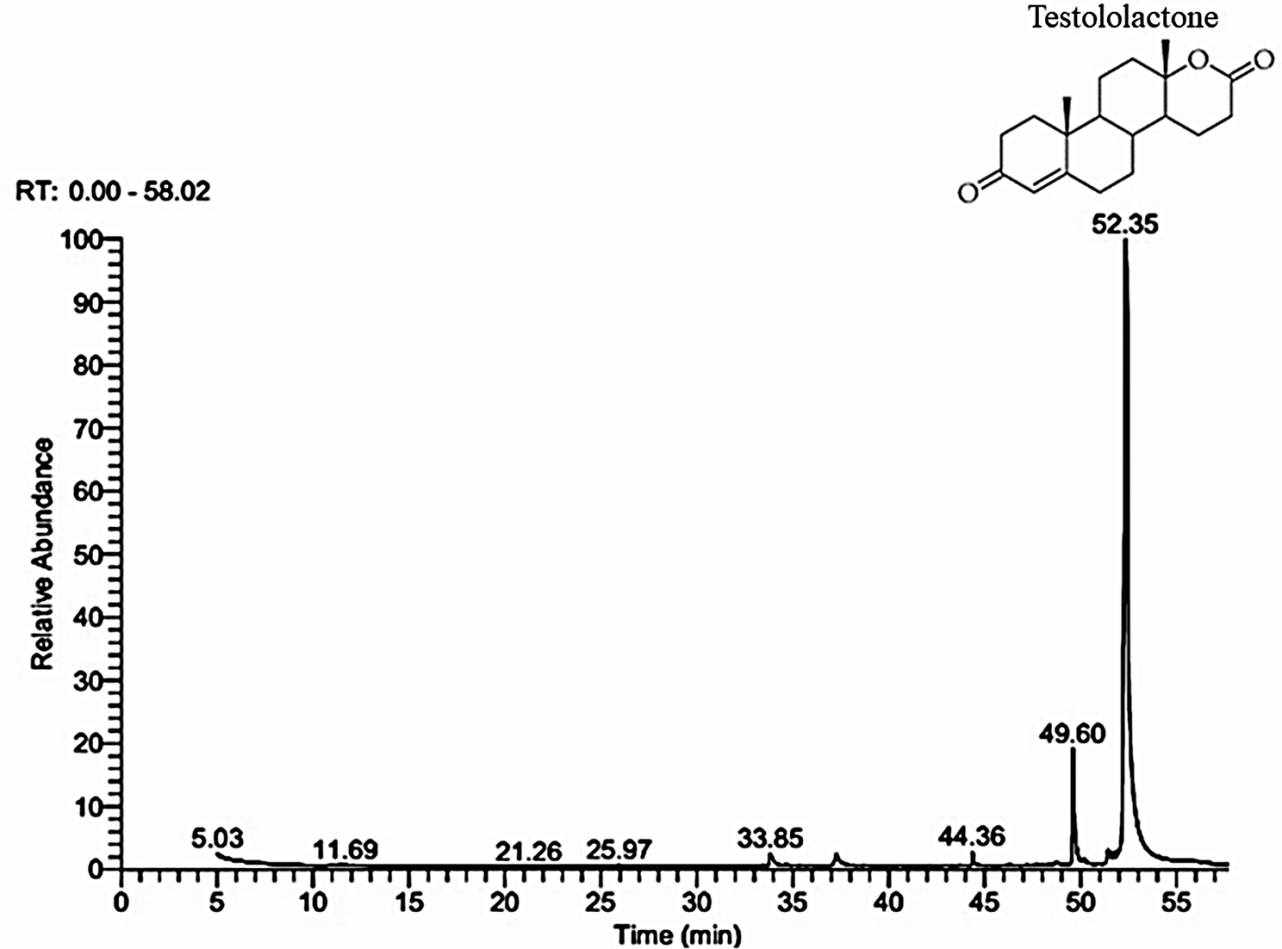
GC–MS spectral chromatograms for progesterone bioconversion by the fungal strain RAS3009

Interestingly, the strain Ras3009 transformed about 89% of progesterone into derivatives, it’s worth noting that almost all of this quantity was converted into a single compound (testololactone). This led us to investigate the identity of the produced compound and raised several questions regarding the potential application of this compound. The GC-MS spectral chart of the chloroform extract of Ras3009 strain (Fig. 2) showed a large peak corresponding to testololactone as the main product results from progesterone biotransformation. The height and area of this peak indicate that testololactone is the most abundant component in the sample, which accounts for approximately 81.18% of the total products detected by GC-MS (Table 1). Minor byproducts produced as secondary metabolites during fungal metabolism include pentadecanoic acid, 14-methyl, methyl ester; 9,12-octadecadienoic acid, methyl ester; and 1,2-benzenedicarboxylic acid, dioctyl ester were detected.

**Table 1:** GC-MS analysis of progesterone transformation by the fungal strain Ras3009

RT (min)	Metabolite	MF	MF	MA (%)
33.84	Pentadecanoic acid, 14 methyl, methyl ester	270	C_17_H_34_O2	1.49 ± 0.19
37.26	9,12 Octadecadienoic acid, methyl ester	294	C_19_H_34_O_2_	1.15 ± 0.07
44.36	1,2 Benzenedicarboxylic acid, dioctyl ester	390	C_24_H_38_O_4_	1.05 ± 0.04
49.60	Progesterone	314	C_21_H_30_O_2_	11.01 ± 1.12
52.34	Testololactone	302	C_19_H_26_O_3_	81.18 ± 1.7

RT; retention time, MW; Molecular weight, MF; Molecular formula, MA (%) Metabolite abundances are mean values from three replicates with associated standard deviations

**Fig.3:**
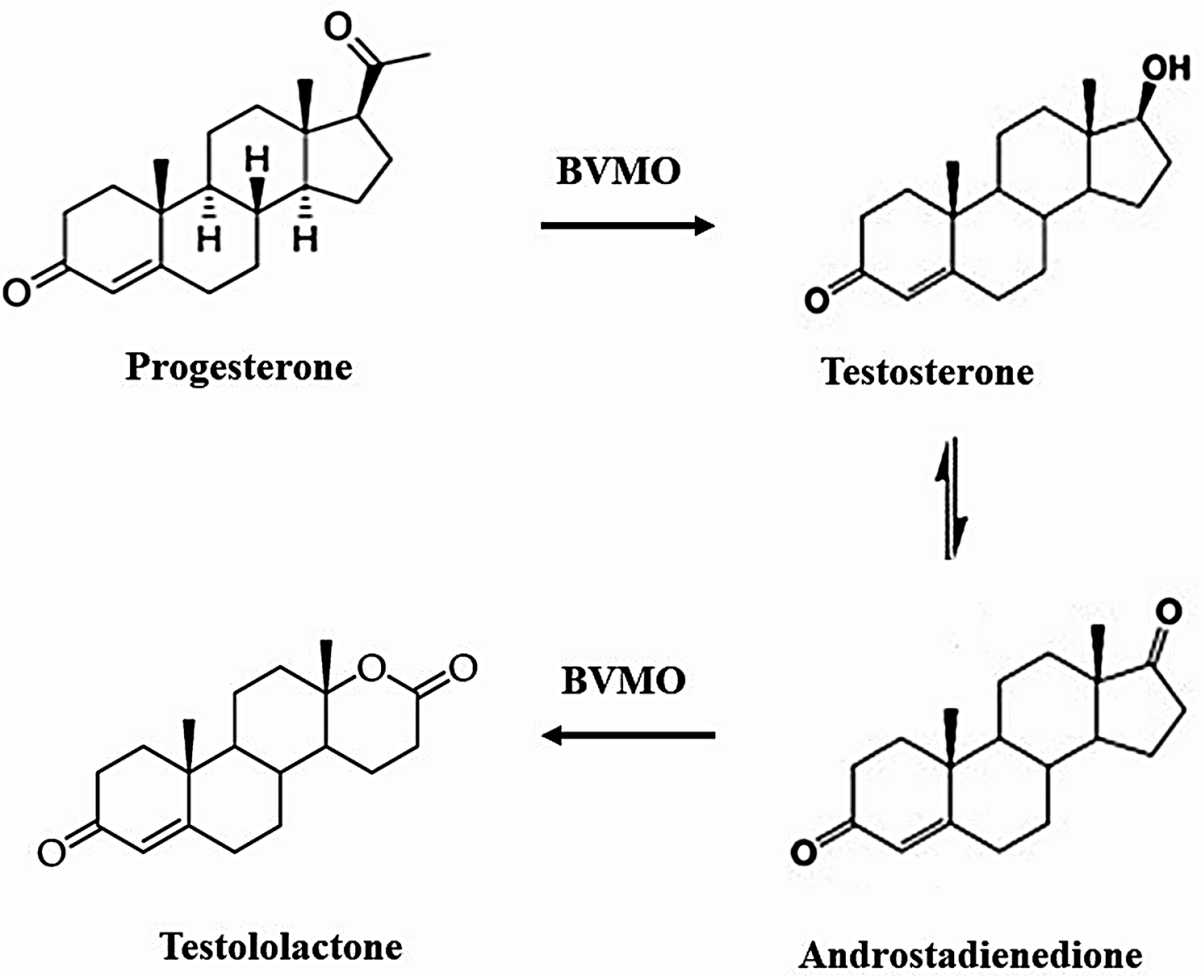
The scheme of the transformation of progesterone into testololactone by the fungal isolate Ras3009

This diagram represented in Fig. 3 illustrates the biochemical pathway of progesterone transformation through various intermediates to testololactone. Progesterone, a 21-carbon steroid, is converted to testololactone through a series of steps mediated by Baeyer-Villiger monooxygenases (BVMOs) in the fungal strain *P. chrysogenum* Ras3009. The pathway begins with 17α-hydroxylation of progesterone, converting it to 17α-hydroxyprogesterone, which still retains the 21-carbon structure. This is followed by cleavage of the side chain, leading to the formation of androgens with a 19-carbon skeleton, particularly testosterone. Testosterone is then oxidized to form androstenedione, a 19-carbon compound with a 3-keto group. The final and critical step is the conversion of androstenedione to testololactone. BVMOs catalyze the insertion of an oxygen atom into the 3-keto group of androstenedione, which results in the formation of a 19-carbon lactone ring in testololactone. This lactone ring is a hallmark of testololactone, distinguishing it from other steroidal compounds.

**Fig.4:**
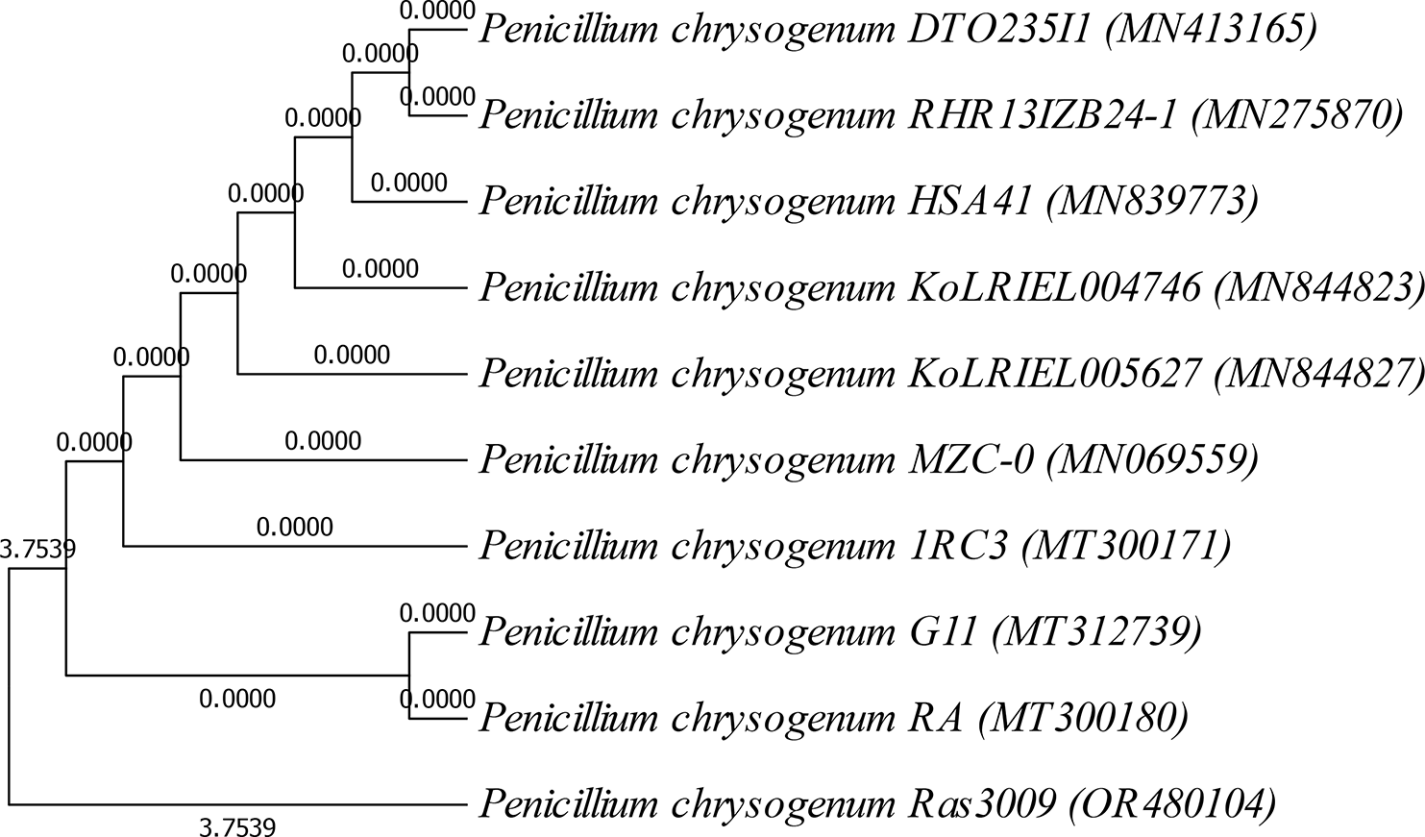
Evolutionary relationships of *Penicillium chrysogenum* Ras3009 with the efficiency of progesterone transformation into testololactone by the intacelleular, extracellular and whole cell *of P. chrysogenum* Ras3009

The selective fungal strain Ras3009 was identified by relying on the phenotypic characteristics and analyzing its genetic makeup. This was achieved by characterizing both the macroscopic and microscopic morphological growth characteristics of the selected fungal isolate Ras3009. In addition, the nucleotide sequence of the amplified 18 S rRNA gene (1100 bp) was examined. The analysis revealed that this strain, Ras3009, is essentially the same as *Penicillium chrysogenum* RA (MT 300180), with a 99% similarity. The position of Ras3009 among other closely related *Penicillium* species is depicted in Fig. 4. The figure presents a tree diagram that is proportional in scale, with the branch lengths representing the evolutionary distances inferred from the genetic data. The evolutionary distances were calculated using the maximum composite likelihood method and are measured in terms of the number of base substitutions per site (Fig. 4). The analysis involved 10 nucleotide sequences, and it considered all codon positions, including 1st, 2nd, 3rd, and noncoding regions. Gaps and missing data were excluded from the analysis. The final dataset consisted of 499 positions. The nucleotide sequence of 18 S rRNA of *Penicillium chrysogenum* Ras3009 has been assigned the accession number OR480104 in the GenBank database.

**Fig.5:**
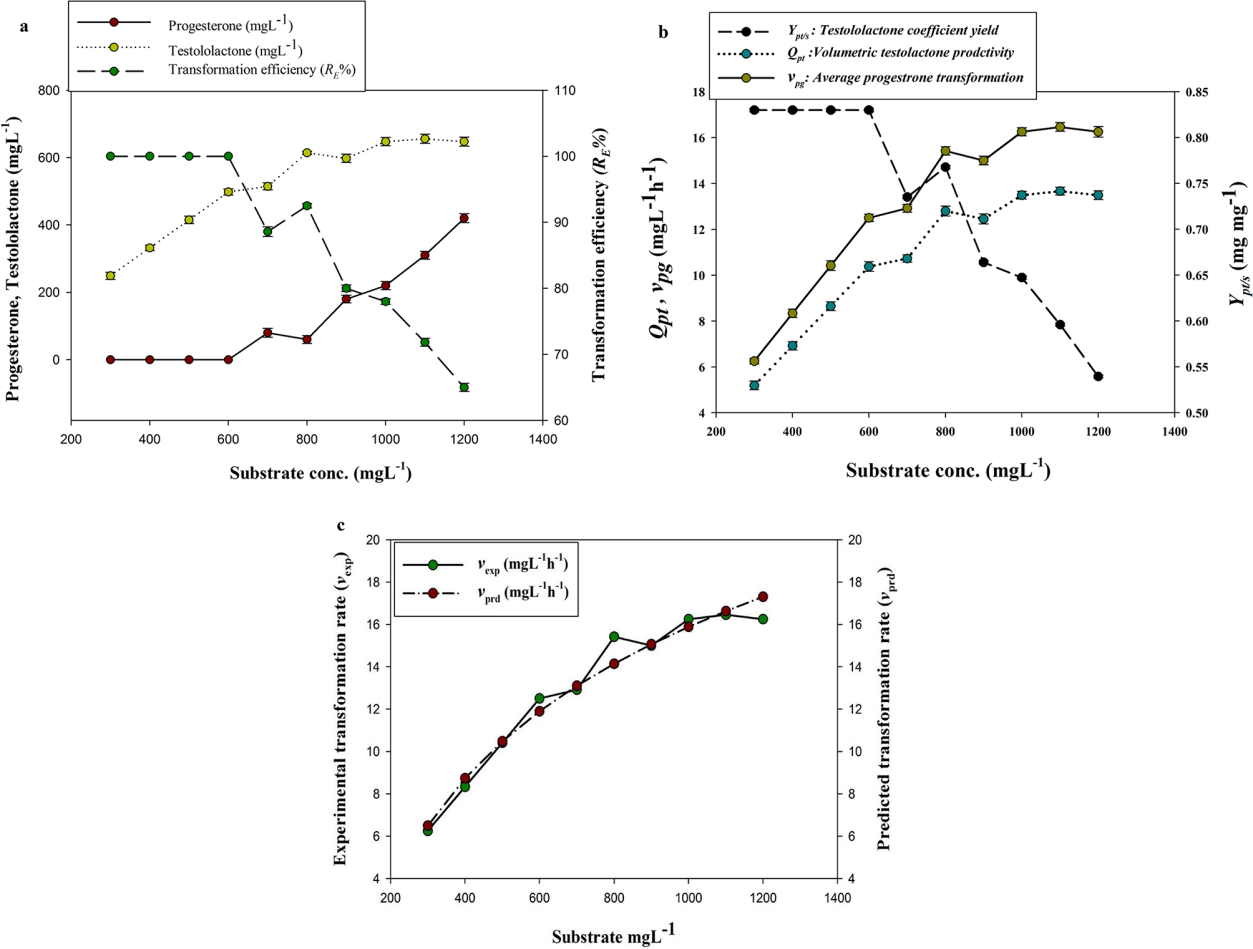
(**a**) Effect of substrate concentration on the biotransformation of progesterone into testololactone by *P. chrysogenum* Ras3009. (**b**) Effect of substrate concentration on testololactone coefficient yield (*Y*_*pt/s*_), volumetric testololactone productivity (*Q*_*pt*_), and average progesterone transformation rate (*v*_*pg*_*)*) by *P. chrysogenum* Ras3009. (**c**) The specific transformation rates (experimental and model predicted) by *P. chrysogenum* Ras3009 at different progesterone concentrations

Progesterone transformation efficiency into testololactone was maximized by studying the impacts of fermentation conditions including the substrate concentration, incubation period, and type of cellular extract on the transformation efficiency parameters. Results indicated that initially there was a positive correlation between the substrate concentration and the testololactone produced, giving the highest testololactone concentration of 655.7 mgL^− 1^ when the progesterone concentration was recorded as 1100 mgL^− 1^ (Fig. 5a). Also, an increase in the volumetric testololactone productivity *(Q*_*pt*_*)* and the average progesterone transformation rate *(v*_*pg*_*)* were noted at the initial progesterone concentrations increased (Fig. 5b). However, statistical analysis showed that the produced testololactone concentrations, testololactone productivity *(Q*_*pt*_*)* and the average progesterone transformation rate *(v*_*pg*_*)* were not significantly changed at the very high progesterone concentrations. Kinetic studies were performed using initial progesterone (substrate) concentrations chosen as independent variables for average progesterone transformation rates of the culture. Data were represented using nonlinear regression by Monod model as follows:

5 $$\begin{gathered}{v_{pg}} = {v_{max}}\frac{{{S_{go}}}}{{{K_s} + {S_{go}}}} \hfill \\{v_{pg}} = 0.0482\frac{{{S_{go}}}}{{19.249 + {S_{go}}}},\,{R^2} = 0.977 \hfill \\ \end{gathered}$$

Where *v*_max_ is the progesterone transformation rate, S_go_ is the initial progesterone concentration, and *K*_s_ is the progesterone transformation constant of the Monod model. It was estimated that *v*_*max*_ was calculated as 0.0482 gL^− 1^hr^− 1^ with high accuracy, giving *R*^2^ values of 0.977. Both the experimental and model predicted specific progesterone transformation rates by *P. chrysogenum* Ras3009 at different progesterone concentrations were represented in Fig. 5c.

**Fig.6:**
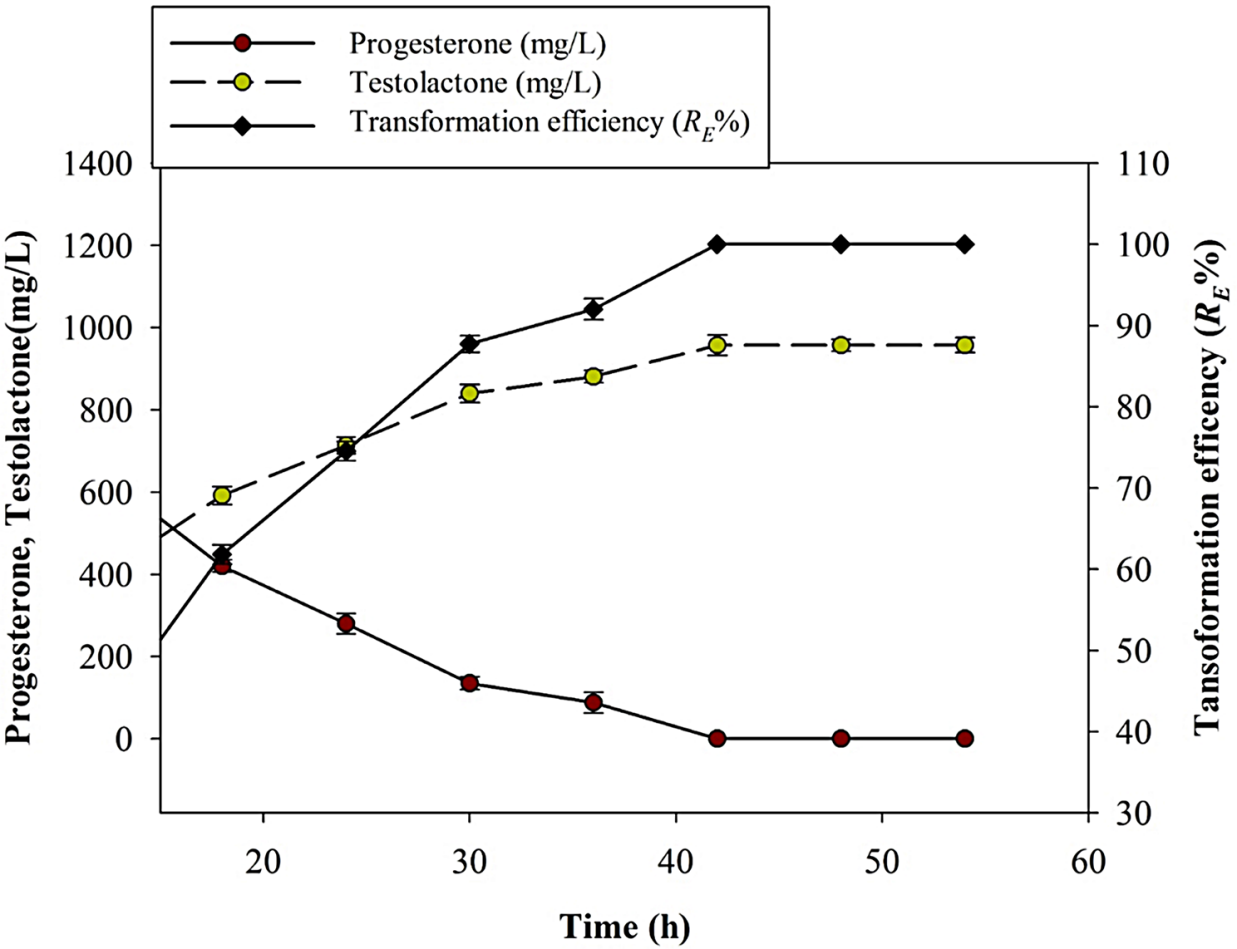
Effect of incubation time on the biotransformation of progesterone into testololactone by *P. chrysogenum* Ras3009

The data in Fig. 6 indicated that as time increases, the spent progesterone concentration decreases, indicating that more progesterone is being converted into testololactone. The transformation efficiency generally increases with time, reaching a maximum of 100% within 42 h. Moreover, the maximum values of the average testololactone yield (*Y*_*pt/s*_) 0.87 mg/mg and the produced testololactone (*P*_*t*_) 957 mg/L were obtained during the same period. Statistical analysis revealed that after this incubation period, no significant change was observed in the volumetric testololactone productivity *Q*_*pt*_ (Table 2). By studying the type of the used fungus extract as shown in Table 2. It appears that the efficiency of progesterone transformation into testololactone increases as the extract type becomes more cellular. The higher progesterone transformation efficiencies were reported when using intracellular or whole microbial cell extracts.

**Table 2:** Effect of incubation time on the different types of cellular extract efficiency, testololactone coefficient yield (Y_pt/s_), volumetric testololactone productivity (Q_pt_), and average progesterone transformation rate (v_pg_) by P. chrysogenum Ras3009.

Time (h)	WHCE (%)	ICE (%)	ECE (%)	*Y*_*pt/s*_ (mg/mg)	*Q*_*pt*_ (%)	*v*_*pg*_ (mgL^− 1^h^− 1^)
12	40.90^a^ ± 1.17	29.21^a^ ± 0.21	8.02^a^ ± 0.07	0.355^a^	32.62^a^ ± 0.19	37.50^a^ ± 0.18
18	61.81^b^ ± 1.20	50.22^b^ ± 0.22	12.15^b^ ± 0.09	0.537^b^	32.86^a^ ± 0.18	37.77^a^ ± 0.17
24	74.54^cb^ ± 1.18	65.11^c^ ± 0.18	14.18^b^ ± 0.08	0.648^c^	29.72^ab^ ± 0.19	34.16^ab^ ± 0.19
30	87.72^cd^ ± 0.10	78.12^cd^ ± 0.19	18.12^c^ ± 0.09	0.763^d^	27.98^abc^ ± 0.21	32.16^ab^ ± 0.18
36	92.00^d^ ± 0.10	82.00^d^ ± 0.22	19.34^cd^ ± 0.08	0.800^d^	24.45^bcd^ ± 0.16	28.11^b^ ± 0.18
42	100.00^d^ ± 0.0	89.00^d^ ± 0.23	21.15^cd^ ± 0.09	0.870^d^	22.78^bd^ ± 0.18	26.19^bc^ ± 0.17
48	100.00^d^ ± 0.0	91.12^d^ ± 0.22	22.45^d^ ± 0.11	0.870^d^	19.93^de^ ± 0.19	22.91^cd^ ± 0.19
54	100.00^d^ ± 0.0	93.00^d^ ± 0.21	23.11^d^ ± 0.11	0.870^d^	17.72^e^ ± 0.17	20.37^d^ ± 0.11

HCE; Whole cell extract efficiency (%), ICE; Intracellular extract efficiency (%), ECE; Extracellular extract efficiency (%), Y^pt/s;^ Testololactone coefficient yield (mg/mg), Q^pt;^ Volumetric testololactone productivity (mgL^-1^h^-1^), Q^s^; Average progesterone transformation, (mgL^-1^h-^1^). Values are means of three replicates; values followed by same letters on the same column are not significantly different (P < 0.005) in Tukey’s test

## Discussion

Progesterone, a naturally occurring steroidal hormone found in female mammals, serves as a key component in contraceptive medications like norethindrone, norgestimate, medroxyprogesterone acetate, levonorgestrel, and desogestrel [30]. Progesterone can be transformed into testololactone, a steroid compound known for its ability to inhibit aromatase enzymes [31]. Testololactone has shown potential activity in the treatment of breast cancer, prostatic hyperplasia, and prostate cancer [32]. Additionally, it can function as a therapeutic agent for disorders resulting from imbalances between estrogen and androgen actions, such as gynecomastia [33] and precocious puberty [24].

The present investigation suggested that certain fungal strains can be efficient catalysts for transforming progesterone, and their use in industrial applications may help optimize the production process. Our findings agree with previous studies that have shown the potential of filamentous fungi in the biotransformation of steroids. For example, Zhang et al. [34] reported that *Fusarium oxysporum* could convert pregnenolone, a precursor to progesterone, into various metabolites. According to Savic et al. [24], steroid biotransformation by microbes offers many advantages over chemical synthesis, such as the high regio-, chemo-, and enantioselectivity and more environmentally friendly processes.

Progesterone conversion to testololactone by fungi might provide evidence of lactone formation in D-ring steroids [24, 35]. Numerous fungi such as *Penicillium chrysogenum*, *Aspergillus flavus*, and *Cylindrocarpon radicola* produce flavoenzymes called Baeyer-Villiger monooxygenases (BVMOs) that catalyze the conversion of ketones to the corresponding lactones or esters via the Baeyer–Villiger reaction [35, 36]. However, few organisms are capable of bio-oxidation, including both dehydrogenation in ring A and lactone formation in D-ring steroids [37]. Javid et al. [38] revealed that the progesterone bioconversion into testololactone by *Aspergillus sojae* is processed through a 3-step pathway including: cleavage of the 17β-acetyl side chain, oxidation of 17β-hydroxylation, and oxygenation of lactones to the 17-ketone indicating BVMO activity in the fungal isolate.

In the present study, *Penicillium chrysogenum* Ras3009 was recorded as an efficient biocatalyst in converting progesterone into testololactone. Some other microbial strains have been reported to transform steroidal compounds to testololactone. For example, *Aspergillus tamari* KITA transformed 17a-hydroxy progesterone and deoxycorticosterone to testololactone [39]. Cardoso de Paula and Porto [40] reported the ability of marine-derived fungus *Aspergillus sydowii* CBMAI 935 to convert the steroid progesterone to testosterone and testololactone. Moreover, Bartmanska et al. [41] studied the use of *Penicillium notatum* for 1-dehydrotestosterone biotransformation and noted that the fungus could insert an oxygen atom into the D-ring and convert it into testololactone with 90% yield upon the chloroform extract.

Also, there were other species of *Penicillium* genus, which could give rise to testololactone. For instance, *Penicillium camembertii* AM83, *Penicillium notatum* and *P. simplicissimum* WY134-2 were recorded to convert progesterone to testololactone [42–44].

Kinetic modelling of experimental studies enables us to predict and evaluate the effect of various conditions and medium components on microbial physiology [45, 46]. A Monod kinetic model can be used to evaluate product formation concerning substrate utilization [47]. It was indicated that progesterone concentration, incubation period, and cellular extract type are crucial factors to maximize testololactone production and to increase its yield. In addition, these factors greatly influenced the kinetic parameters during the transformation process.

In the present investigation, the substrate concentration (progesterone) directly affected the biotransformation of progesterone into testololactone and the spent progesterone amount. Similarly, Yang et al. [43] reported that testololactone yields increased by increasing the progesterone concentration and reached up to 96% yield using 3 g/L progesterone. However, it was revealed that as the substrate concentration increases, the spent progesterone also increases, so the transformation percent decreases. This implies that a very high substrate concentration may reduce the efficiency of the transformation reaction and slightly lower the average testololactone yield (*Y*_*pt/s*_). The reason might be that the positive impact of increasing the substrate concentration on the reaction rate starts to decline until a stage where little further impact exists. This point indicates the maximum velocity *v*_*max*,_ at which the catalytic enzyme is almost saturated with the substrate [48].

By studying the effect of different incubation periods on the transformation efficiency and testololactone production during the enzymatic conversion of progesterone into testololactone. This suggests that the transformation rates of the catalytic enzymes in converting progesterone into testololactone reach the maximum after 42 h. Javid et al. [38] reported that the maximum amount of testololactone resulting from the biotransformation of progesterone by *Aspergillus sojae* PTCC 5196 was attained during 24 to 28 h.

The data obtained in the present study also assumed that Bayer-Villier monooxygenases (BVMOs) may be the catalytic enzymes for the transformation by *P. chrysogenum* Ras3009, and are expressed intracellularly, so degrading fungal cells is necessary to achieve high transformation efficiency. These findings agreed with Li et al. [48] who revealed that cyclohexanone monooxygenase (CHMO), which is characterized as type I BVMOs, is produced and expressed intracellularly, and a cell-breaking is needed to achieve high-quality and large-scale industrial application.

## Conclusions

The work demonstrated that the fungal isolate *P. chrysogenum* Ras3009 is an efficient biocatalyst for converting progesterone into testololactone with a transformation efficiency up to 100%. It was indicated that progesterone concentration, incubation period, and cellular extract type are crucial factors in maximizing testololactone production and increasing its yield. In addition, these factors greatly influenced the kinetic parameters during the transformation process. Bayer-Villier monooxygenases (BVMOs) may be the catalytic enzymes for the transformation of *P. chrysogenum* Ras3009, and are expressed intracellularly, so degrading fungal cells is necessary to achieve high transformation efficiency.

## Data Availability

18 S rRNA nucleotide sequence of the fungal isolate P. chrysogenum Ras3009 was deposited in the GenBank database and assigned the accession number OR480104. All the analyzed and generated data are included in this study.

## Abbreviations

BVMOs: Bayer-Villier monooxygenases
CHMO: Cyclohexanone monooxygenase
*R*_*E*_: Transformation efficiency
*P*_*t*_: Testololactone concentrations
*Y*_*pt/s*_: Testololactone coefficient yield
*Q*_*pt*_: Volumetric testololactone production rate
*v*_*pg*_: Average progesterone transformation rate
*S*_*nt*_: Non-transformed progesterone
*S*_*t*_: Progesterone transformed
*S*_*go*_: Initial progesterone concentration
